# The New Generation of Professional Soccer Talent Is Born under the Bias of the RAE: Relative Age Effect in International Male Youth Soccer Championships

**DOI:** 10.3390/children8121117

**Published:** 2021-12-02

**Authors:** Benito Pérez-González, Jairo León-Quismondo, José Bonal, Pablo Burillo, Álvaro Fernández-Luna

**Affiliations:** 1 Faculty of Business and Communication, Universidad Internacional de La Rioja, 26006 Logroño, Spain; benito.perez@unir.net; 2 Faculty of Sports Sciences, Universidad Europea de Madrid, Calle Tajo S/N, Villaviciosa de Odón, 28670 Madrid, Spain; jose.bonal@universidadeuropea.es (J.B.); pablo.burillo@universidadeuropea.es (P.B.); alvaro.fernandez2@universidadeuropea.es (Á.F.-L.)

**Keywords:** Relative Age Effect, youth soccer, birthdate, selection bias, youth development

## Abstract

In 2019, numerous championships of youth categories soccer national teams were held. In the present study, we analyzed the existence of the Relative Age Effect (RAE) in four major male championships that, due to their importance and participating teams, most likely brought together the great bulk of the players who will dominate professional soccer in the next decade. Participants were professional and amateur youth male soccer players who participated in the last international championships: UEFA European Under-21 Championship (2017–2019); UEFA European Under-19 Championship (2019); South American Youth Football Championship (also known as Conmebol U-20) (2019); and FIFA U-20 World Cup (2019), with 823 players (20.25 ± 0.84 years). In the four championships analyzed, the existence of RAE was found for all players (*p* < 0.001). Analyzing the players when considering their position on the pitch and their championship, RAE was found, statistically significant, in 10 of the 16 classifications. New generations of elite soccer players arrive with a clear bias in the selection of talent; an unfair bias, based on unequal opportunities in early categories, which should be reviewed by sports authorities.

## 1. Introduction

Talent development is a current concern in most sports organizations, struggling to create adequate career paths for players or athletes from the grassroots. In most sports institutions, the organizational strategy at early ages for grouping players or athletes follows a cutoff criterion, similar to most of the school systems [1]. This means that players are grouped according to their date of birth instead of their physical or psychical development. The differences in growth and maturity of players result in disparities of performances, opportunities, and, consequently, unequal presence in competitions according to their age and birth month. This effect of asymmetry in the birth distribution is known as the Relative Age Effect (RAE) [2]. It is an extensively studied bias descriptor that reveals the existence of a lower number of players born in the last months close to the cutoff. In sports, the cutoff is set as 1st of January in most of the competitions, so players born in the firsts months of the year have improved prospects in sports [3].

The RAE was first questioned by Grondin et al. [4] and explored shortly after by Barnsley et al. [5]. Since then, this bias effect has been extensively studied in different sports, including team sports such as soccer [6], basketball [7,8], hockey [9], rugby [10,11], futsal [12], handball [13], baseball [14], Australian football [15] water-polo [6], and volleyball [6]; and individual sports like athletics [16,17,18], alpine ski racing [19], swimming [20,21], tennis [22], and even golf, horse racing, sumo, or badminton [23]. As a consequence, there is a large number of articles that have led to diverse literature reviews and meta-analyses that try to make more accessible all this information [1,24].

One of the sports that has received more attention on RAE is soccer [1]. It is one of the most explored sports regarding asymmetry in the birth distribution, with fewer players born in the last quarter of the year [12]. Researchers have focused on diverse leagues from countries such as the United States [25], Argentina [3], or China [26]. In Europe, this interest has led to work in specific countries and national leagues [27], proving that this phenomenon is present in the top five European leagues (e.g., Premier League, LaLiga, Bundesliga, Serie A, and Ligue 1). This includes research on England [28], Spain [29,30], Germany [31], Italy [32], and France [33]. RAE has also been explored in international competitions such as the Union of European Football Associations (UEFA) Championship or the Fédération Internationale de Football Association (FIFA) competitions. For example, a work by González-Víllora et al. [34] studied Under-17, Under-19, and Under-21 elite soccer players in the UEFA Champions League, confirming that RAE was present in those categories. Another example is the case of FIFA competitions, in which Saavedra-García et al. [35] established that RAE exists in male FIFA competitions and its effect is dynamic and complex.

This disparity of distribution of players according to participants’ age is especially critical for youth. Physical and conditioning variables seem to be the causes for higher chances of older players being chosen in the selection process in comparison to younger ones [12]. In soccer, the multifactorial nature of the sport, where many diverse factors interact (anthropometric, body composition, somatotype, physical and physiological factors, or soccer-specific skills) makes it even more difficult to detect new talents at early ages [36,37]. RAE is present in all the stages of the players’ careers. In this regard, some research suggests that the more the competitive level, the higher RAE, since the selection process is more demanding [38]. However, there is also contrasting evidence that RAE influence is greater on younger age categories [34]. In addition, male categories seem to have more RAE. For example, a study conducted by Smith et al. [39] proved that RAE in female soccer is not as powerful as in male categories. Baker et al. [40] observed in their research that gender acts as a moderator of RAE. As can be seen, male soccer continues suffering from unequal representativeness of players by month of birth. Therefore, further information is needed for shedding light on the talent identification process.

With all this background, researchers and practitioners have tried to offer some solutions. One of the proposals for reducing the RAE is bio-banding, consisting of grouping players according to other variables apart from age, such as physical maturity [41]. In this line, recent work has proved this initiative to be efficient for limiting RAE [42,43].

Although the RAE was first reported more than 30 years ago, no structural or organizational changes have been produced for limiting RAE over the past 10 years in soccer. Many soccer fans or authorities should ask themselves: In the best youth soccer competitions, do the best possible players come? Or do the best players from an inefficient talent selection come? Since this bias is critical for equal talent identification, a better understanding would help managers, athletes, sports systems, and sport policymakers to avoid this phenomenon internationally. Thus, the purpose of this study is to examine the birth dates distribution and RAE by position and competition of professional/amateur youth male soccer players who participated in four recent international championships in different geographical regions: UEFA European Under-21 Championship (2017–2019), UEFA European Under-19 Championship (2019), South American Youth Football Championship (2019), and FIFA Under-20 World Cup (2019). This study allows covering youth soccer from an international perspective, addressing some of the most important worldwide soccer competitions.

## 2. Materials and Methods

### 2.1. Participants

Participants in the four aforementioned competitions, UEFA European Under-21 Championship (2017–2019); UEFA European Under-19 Championship (2019); South American Youth Football Championship (also known as Conmebol U-20) (2019); and FIFA U-20 World Cup (2019), were studied (*n* = 823) using official UEFA and FIFA competitions data. The average age of the players was 20.25 ± 0.84 years.

### 2.2. Procedure and Data Analysis

The Relative Age Effect (RAE) was detected through Poisson regression [44,45]. The Poisson regression formula y = e (b0 + b1x) serves to explain the frequency count of an event (y) by an explanatory variable x. The data used for Poisson regression were the week of birth (WB), whereby the first week in January was designated WB 1, and time period of birth (tB), describing how far from the beginning of the year a player was born. This last index ranging between 0 and 1 was calculated as tB = (WB − 0.5)/52.

In the Poisson regression, the event (y) was the frequency of birth in a given week and the explanatory variable (x) was tB. We also calculated the index of discrimination (ID) according to Doyle and Bottomley [45] as e-b1. This index measures the relative odds of a player born on day 1 versus day 365 of the competition year being selected. The likelihood ratio R2 was determined according to Cohen et al. [46]. All statistical tests, including descriptive analysis, were performed using the software package SPSS-v20 (Statistical Package for Social Science, IBM Inc., Chicago, IL, USA). Significance was set at *p* < 0.05.

## 3. Results

Birthdate distributions by quartile (Q) and semester (Se) for the players in the different championships are shown in Table 1.

**Figure 1:**
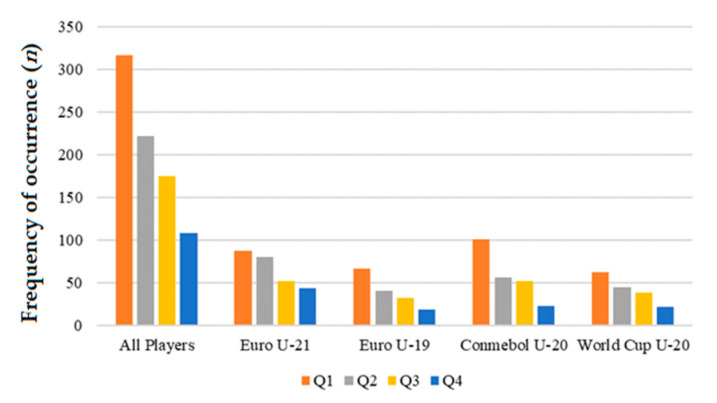
Quartile of birth of players by championship.

In Figure 1, the predominance of the first quartiles over the following is visually perceived. Poisson regression by frequency analysis revealed the presence of a significant overall RAE across all championships analyzed. The effect was not significant for goalkeepers in the championships analyzed, except for Euro U-21 (*p* < 0.001). However, for other positions, RAE was significant in most championships including defenders participating in Euro U-19, Conmebol U-20, and World Cup U-20; midfielders in Euro U-21, Euro U-19, and Conmebol U-20; and forwards in Euro U-19, Conmebol U-20, and World Cup U-20 tournaments (Table 2).

**Figure 2:**
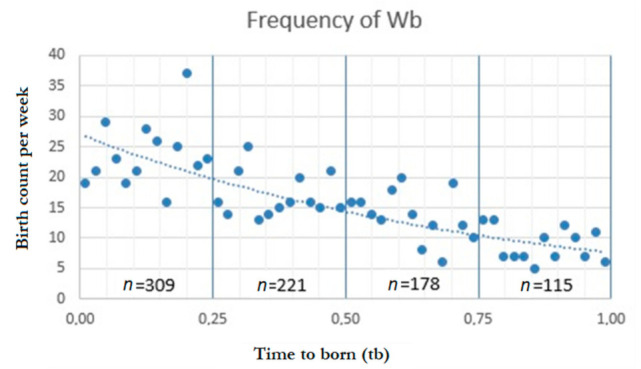
Poisson regression fit line regarding the frequency of week of birth (W_B_).

Figure 2 represents the Poisson regression fit line of the total number of players for each week of birth (Wb) and divided by quartiles.

## 4. Discussion

The first results show a high RAE effect for all the U19, U20 and U21 competitions analyzed in the present study independently of the geographical region (Europe, America, or World level competitions). In similar methodological studies targeting Asian U18, U20 male competitions, and U16-U20 female competitions also the presence of the RAE effect was also found having been studied over 1700 players [26], therefore we could say that this is a global factor that affects all types of sport competitions.

As is already established in the scientific literature, the RAE can play a crucial role for young athletes, allowing them to be selected on sportive youth talent programs [38,47] or later on in their careers, facilitating for them a more relevant role during youth competitions, especially regarding different player development aspects [37]. Athletes have been proven to have prolonged involvement in those sports where they are successful [48]. Therefore, the physical and biological advantages that the RAE effect provides to Q1 and Q2 athletes will facilitate their adherence to the sport for longer time periods and ultimately better opportunities to reach the elite footballer status [49]. Other sport discipline studies on the trajectories of elite tennis players [50] and elite basketball players [51] mentioned that there is an increase in the level of players when they play against rivals of a level equal to or higher than theirs. This is important to originate sports opportunities where the best players can play against each other so that the improvement curve grows as much as possible. However, in fact, coaches are often focused on winning matches instead of promoting the development of talent. Some researches highlight how coaches tend to take decisions more to win the next match than to grow up valuable players in the future [52,53].

More S1 players will enjoy such opportunities since they are predominantly picked to conform to the national team that participates in continental and intercontinental tournaments. Furthermore, player development progression will be positively affected for those athletes participating in the studied competitions, specifically regarding their psychological traits and psychological characteristics development [51] and potentially creating a development gap distance between S1 and S2 born footballers. In a certain way, it is also related to the descriptor by Kristiansen et al. [54], who highlighted the growth as athletes and the acquisition of certain psychological benefits to applying in subsequent “big appointments”, by the athletes participating in the Youth Olympic Games [54], with these potential benefits acquired in big events (e.g., junior world championship).

Having a deeper look into the results section, the findings regarding the relationship between player position and RAE effect confirms the presence of RAE in almost all studied tournaments (except Euro U21) for two of the most determining soccer positions: goalkeeper and forward. Aligning with our results, previous studies with large samples have also connected birthdate and position finding strikers to be the predominant positions with more RAE presence in comparison to defenders or midfielders [26,55] (it is worth considering the limited number of participating goalkeepers at each tournament). This may be due to the great influence of these field positions on the final match result.

Reflecting on the future of the young players participating in youth sports tournaments, it is interesting to highlight that in the most important senior European professional football leagues, an RAE effect is observed [56,57,58]. It is important to point out that these competitions are nurtured by younger players who compete or will compete in the near future at the analyzed tournaments by the present investigation.

Different possible solutions to this RAE effect were presented in the review by Webdale et al. [59], where some structural measures were highlighted, such as limiting the number of players belonging to each quartile in the total workforce [5,60], delimiting the chronological bands of participation [1,61,62] or even using numbers that help to identify older players [63].

Specifically, a particularly interesting proposal to solve this RAE effect (due to its characteristics and the fact that it has been implemented experimentally), was presented by Cumming et al. [43], who used the technique known as bio-banded (grouping of the different teams by the degree of biological-sport maturation, replacing the classic chronological age system) with soccer players in the United Kingdom. All participants described the experience as positive, highlighting aspects such as physical demand and technical–tactical adaptation needs (by the early maturing group), as well as the presence of greater opportunities to demonstrate their skills and competencies (by the late-maturing group) [43]. This bio-banded practice has also been recommended by recent important articles, such as Till and Baker [64].

The new generation of professional soccer talents is born under the bias of the RAE. A bias that is probably unconscious during the selection of talent, but that curtails equal opportunities derived solely and exclusively from a political decision (modifiable then): to divide the lower categories from 1st of January. At the moment, they are all merely theoretical proposals, none of these proposals having been implemented in official competition [59], but in order to reduce the RAE effect for Q3 and Q4 born athletes during their development, it would be a good recommendation to start implementing strategies that build a competition system which provides fairer and equal opportunities to all athletes regardless of their date of birth.

## 5. Conclusions

This study confirms the existence of RAE in youth soccer competitions, regardless of the geographical region. A significantly higher presence of field players benefiting from RAE is observed across most of the championships analyzed (UEFA European Under-21 and Under-19 Championships, Conmebol Under-20, and FIFA Under-20 World Cup). However, in goalkeepers, this effect is significant only in the UEFA European Under-21 tournament.

This article stresses the existing bias depending on the birthdate of soccer players. Although the conclusions are indeed limited to the characteristics of the sample and the sport modality (soccer), coaches and managers of youth clubs should consider the RAE effect for reducing partiality when selecting and developing new talents.

## Figures and Tables

**Table 1:** Birthdate distributions of international youth championships (2017–2019) according to their quartile or semester of birth.

Category	Measure	Q1	Q2	Q3	Q4	Se1	Se2
Euro U-21	*n*	87	80	52	44	167	96
	%	33.1	30.4	19.8	16.7	63.5	36.5
Euro U-19	*n*	67	41	32	19	108	51
	%	42.1	25.8	20.2	11.9	67.9	32.1
Conmebol U-20	*n*	101	56	52	23	157	75
	%	43.5	24.1	22.5	9.9	67.6	32.4
World Cup U-20	*n*	62	45	39	22	107	61
	%	36.9	26.8	23.2	13.1	63.7	36.3
All players	*n*	317	222	175	108	539	28.3
	%	38.6	27.0	21.3	13.1	65.6	34.4

Note: *n* = number; Q = quartile; Se = Semester.

**Table 2:** RAE data analysis by position and tournament.

Position	Measure	Euro U-21 (*n* = 263)	Euro U-19 (*n* = 159)	Conmebol U-20 (*n* = 233)	World Cup U-20 (*n* = 168)
Overall (*n* = 823)	W_B_	23 ± 15	20 ± 14	20 ± 14	22 ± 14
	t_B_	0.43 ± 0.28	0.38 ± 0.27	0.38 ± 0.27	0.41 ± 0.27
	b_0_	2.015	1.751	2.143	1.669
	b_1_	−0.849	−1.436	−1.461	−1.090
	ID	2.34	4.20	4.31	2.97
	R^2^	0.76	0.59	0.48	0.72
	*p* value	<0.001	<0.001	<0.001	<0.001
Goalkeeper (*n* = 107)	W_B_	16 ± 12	27 ± 13	23 ± 15	21 ± 15
	t_B_	0.30 ± 0.24	0.52 ± 0.26	0.44 ± 0.30	0.39 ± 0.29
	b_0_	0.707	−1.274	−0.167	−0.174
	b_1_	−2.741	0.188	−0.748	−1.347
	ID	15.50	0.83	2.11	3.85
	R^2^	0.76	0.99	0.95	0.85
	*p* value	<0.001	0.829	0.236	0.068
Defender (*n* = 266)	W_B_	24 ± 14	19 ± 14	19 ± 13	22 ± 13
	t_B_	0.45 ± 0.26	0.37 ± 0.27	0.36 ± 0.25	0.41 ± 0.25
	b_0_	0.725	0.671	1.134	0.621
	b_1_	−0.592	−1.694	−1.724	−1.087
	ID	1.81	5.44	5.61	2.97
	R^2^	0.94	0.70	−0.65	0.85
	*p* value	0.127	<0.001	<0.001	0.019
Midfielder (*n* = 277)	W_B_	23 ± 15	21 ± 14	20 ± 13	24 ± 15
	t_B_	0.44 ± 0.28	0.39 ± 0.27	0.38 ± 0.24	0.44 ± 0.28
	b_0_	0.973	0.732	0.915	0.290
	b_1_	−0.699	−1.365	−1.434	−0.698
	ID	2.01	3.92	4.20	2.01
	R^2^	0.91	0.79	0.73	0.94
	*p* value	0.047	0.004	<0.001	0.159
Forward (*n* = 173)	W_B_	25 ± 16	18 ± 14	20 ± 16	20 ± 14
	t_B_	0.47 ± 0.30	0.34 ± 0.27	0.38 ± 0.31	0.38 ± 0.27
	b_0_	0.065	0.450	0.751	0.263
	b_1_	−0.342	−2.022	−1.551	−1.501
	ID	1.41	7.55	4.72	4.49
	R^2^	0.98	0.65	0.73	0.78
	*p* value	0.500	0.002	0.002	0.015

## Data Availability

The data for this study is available on Euro U-21 https://es.uefa.com/under21/ (accessed on 14 April 2021); Euro U-19 https://es.uefa.com/under19/ (accessed on 14 April 2021); Conmebol U-20 https://www.livefutbol.com/equipos/ecuador-u-20-h-team/u20-h-campeonato-sudamericano-2019-chile/2/ (accessed on 14 April 2021); World Cup U-20 https://www.fifa.com/es/tournaments/mens/u20worldcup/fifa-u-20-world-cup-poland-2019 (accessed on 14 April 2021). Specific data can be obtained by contacting the lead author B.P.-G.
